# Eco-Innovation in the Food Industry: Exploring Consumer Motivations in an Emerging Market

**DOI:** 10.3390/foods13010004

**Published:** 2023-12-19

**Authors:** Katherine Mansilla-Obando, Gonzalo Llanos, Esteban Gómez-Sotta, Paulo Buchuk, Francisco Ortiz, Mario Aguirre, Fabian Ahumada

**Affiliations:** 1 Faculty of Economics and Business, Universidad Finis Terrae, Santiago 7501015, Chile; gllanos@uft.cl (G.L.); egomez@uft.cl (E.G.-S.); pbuchuk@uft.cl (P.B.); maguirrev@uft.edu (M.A.); fahumadac@uft.edu (F.A.); 2 Center of Economics and Sustainability, Universidad Finis Terrae, Santiago 7501015, Chile; 3 Faculty of Education and Social Sciences, Universität Vechta, 49377 Vechta, Germany

**Keywords:** consumers, sustainable development, eco-innovation, food industry, theory of planned behavior

## Abstract

The utilization of eco-innovative products has witnessed a surge in adoption, driven by their inherent capacity to address pressing environmental concerns. To comprehensively fathom the underlying motivations propelling consumers to embrace these products, we conducted an in-depth investigation employing “The Not Company” (Chile) as a compelling case study. We conducted qualitative interviews with a cohort of 20 Chilean consumers, guided by the Theory of Planned Behavior theoretical framework. The research methodology harnessed the principles of thematic analysis, yielding insights that underscore the significance of key determinants in shaping consumers’ choices towards eco-innovative products. Specifically, our findings highlighted that consumer choices in this domain are profoundly influenced by their attitudes, subjective norms, and perceived behavioral control. Moreover, within these overarching categories, we unearthed sub-themes illuminating the intricate influences guiding consumer choices. These sub-themes encompassed beliefs about food manufacturing and packaging, the persuasive impact of social media and advertising, and the indelible impressions left by prior encounters with eco-innovative products. This study highlights consumers’ fundamental role in the broader eco-innovation landscape, particularly within the food industry context.

## 1. Introduction

In today’s world, taking measures to diminish the planet’s deterioration is imperative. Fortunately, innovative solutions are available to consumers who want to contribute to this cause. In fact, in 2015, the United Nations (UN) established Sustainable Development Goal (SDG) number 12, also known as SDG 12, intending to ensure sustainable consumption and production patterns. It is considered one of the critical goals for development and success [1,2]. Additionally, UN SDG 3 aims to ensure healthy lives and promote well-being for all individuals of all ages. UN SDG 2 seeks to ensure the end of hunger, achieve food security and improved nutrition, and promote sustainable agriculture. SDG 12, SDG 3, and SDG 2 are targets to be achieved by 2030, as set by the UN [1,3].

As a result, the food industry has undergone a significant revolution by embracing sustainability and environmental responsibility. The focus is on caring for the environment through responsible practices, from extracting natural resources for food manufacturing to promoting conscious consumption [4]. This way, the food industry tries to ensure environmentally friendly processes in producing certain types of food, especially those related to the agri-food industry, which significantly demands finite or slow-recovering natural resources, such as freshwater.

Among the sustainable practices adopted by firms, eco-innovation is recognized as one of the strategies that allows them to enhance performance and competitiveness while facing environmental issues and responding to social and regulative pressures [4,5,6,7]. Eco-innovation, as defined by the Organization for Economic Cooperation and Development (OECD), refers to “the development of new or improved products (goods and services), processes, marketing methods, organizational structures, and institutional arrangements that intentionally or unintentionally lead to a reduction in environmental impacts compared to relevant alternatives” [8] (p. 2). However, firms’ investment in and development of eco-innovation practices are insufficient. It is crucial to find a balance between market demands and consumer preferences to ensure the success of ecological products in the market [7,9] through sustainable consumption. Sustainable consumption describes consumers’ attitudes and behavior toward sustainability. It represents a “new way of life” that seeks the sustainable consumption of environmentally friendly products and resources in a responsible way to preserve the environment and reduce negative impacts. 

Environmentally friendly products are based on environmental protection and sustainable development, considering the choice of materials for production in compliance with environmental protection standards [10,11]. This concept has been studied and associated with consumers’ purchase intention; however, the food industry is experiencing increasing innovation and has been rapidly evolving, especially in incorporating new food techniques through technology in food production [2].

Therefore, this study mainly aims to advance the literature on eco-innovation by adopting a consumer perspective and exploring the drivers that lead consumers to purchase and consume eco-innovative products in the food industry in emerging markets. This knowledge is relevant for scholars and practitioners alike since, as indicated by Auestad et al. [12], plant-based alternatives or animal-based substitutes are considered environmentally sustainable, as these foods can have a reduced negative impact on the environment. An example of these products are plant-based drinks, also known as non-dairy or alternative drinks; they refer to drinks that have unique characteristics in flavor, texture, and nutritional composition, which include nuts, cereals, legumes, and seeds, as well as unique processing technologies, which offer consumers a wide range of options [13,14]. Furthermore, these type of products still have low standards, with more insufficient regulation in emerging markets; their prices are often significantly higher than traditional products, their market shares remain limited, and consumers are not always up to date about the new tendencies [2]. 

To achieve the objectives of this research, we selected the firm “The Not Company”, also known as “NotCo”. This Chilean Unicorn, valued at USD 1.5 billion (Forbes (2022). Retrieved from: https://goo.su/xOzHC7, accessed on 11 July 2023), specializes in producing plant-based foods using artificial intelligence (AI), creating alternatives to animal-based products that have similar texture, taste, and color but are made with natural ingredients. After its successful entry into international markets such as Argentina, Brazil, the United States, Canada, and, recently, Mexico (Financial Times (2022). Retrieved from: https://bit.ly/43hGtih, accessed on 3 November 2023), NotCo becomes an ideal company to use for examining and understanding the motivations driving the consumption of eco-innovative products in the food industry, especially in an emerging economy where cultural aspects and environmental behavior can play a significant role in consumer decisions [2]. 

While previous research shows different drivers toward consumer decisions to opt for eco-friendly product, including age, environmental consciousness, perception of quality, and price perception [15], there is a lack of research on the motivations behind the consumption of eco-innovative products in the food industry. Therefore, we pose the following research question: Why do consumers choose eco-innovative products in the food industry? We base our research on the Theory of Planned Behavior (TPB) by Ajzen [16] and the literature on eco-innovation and sustainable development. We explore this conceptual model, which indicates that attitudes toward performing a behavior, social norms, and perceived behavioral control influence behavioral intentions, which then affect actual behavior. To better understand what may drive consumers to purchase eco-innovative products, we explore the model using a qualitative methodology and conducting semi-structured interviews with 20 NotCo consumers, which are analyzed using the thematic analysis proposed by Braun and Clarke [17].

This study contributes to the literature on the consumption of eco-innovative products in a particular company based on the TPB model. It explores why eco-innovative products are purchased in an emerging country like Chile, which has been widely acknowledged as a representative sample of emerging markets [18,19]. Exploring and understanding these reasons for consumption can directly support the company under study, governments, and policymakers to identify and implement strategies to encourage support or not for the consumption of eco-innovative companies.

### 1.1. Sustainable Development and Eco-Innovation in the Food Industry

The profound environmental changes witnessed by society have intensified the debate at national and international levels regarding the urgent need to transition towards development models that strengthen the environmental dimension [20]. In recent years, the concepts of green growth, sustainable economy, and industry have gained increasing prominence, highlighting the importance of changing consumption and production patterns to drive economic growth and ensure environmental sustainability; at the same time, companies continue to promote competition in terms of product quality, prices, and efficient supply chains [21]. In this context, how companies and productive sectors work and produce becomes critical in determining their ability to innovate, improve productivity, and compete in the international market. Creating new production capacities and adopting sustainable technologies represent one of the main challenges for Latin American countries [22].

Sustainability has been a central element in marketing campaigns for many years [23,24]. Conscious consumers have shown a willingness to pay for sustainable products. The case of sustainable development involves setting limits, not absolute limits, but restrictions imposed by the current state of technology and social organization about environmental resources and by the biosphere’s capacity to absorb the effects of human activities [25]. Furthermore, to achieve sustainability, the UN established the 17 SDGs in 2015 as targets to be achieved by 2030. These goals serve as guidance for countries and associated businesses, such as SDG 12, which aims to ensure sustainable consumption and production patterns; SDG 3, which aims to ensure healthy lives and promote well-being for all people at all ages; and SDG 2, which seeks to ensure the end hunger, achieve food security and improved nutrition, and promote sustainable agriculture [1].

While there are various strategies to advance sustainable development, available technology plays a fundamental role in achieving success in developing eco-innovations [26]. Therefore, technology is relevant as it is an indispensable resource for creating and designing new metrics that enable the reform of activities in the interest of environmental care and reducing the associated impact of marketed products and related activities [27].

Eco-innovation is fundamental for organizations’ sustainability and economic growth [5,6,7]. It refers to the production, assimilations, or exploitation of a product, and the production processes, services, management methods, or business models that are novel to the organization and result, throughout their life cycle, in a reduction in environmental risk, pollution, and other negative impacts of resource use (including energy use), compared to relevant alternatives [28]. Implementing eco-innovation can generate economic benefits, although they may not be direct and may vary depending on the type of innovation and the context in which it is used [29]. For example, studies on per capita Gross Domestic Product (GDP) in China analyzing the profitability of environmental policies show that green projects are among the most profitable, projecting an increase in GDP from 0.03% in 2020 to 0.17% in 2030 but identifying high strategic barriers such as financing [30].

In the food industry, eco-innovation is particularly relevant due to the significant direct and indirect environmental effects of food production and consumption [31]. Companies in this sector heavily rely on natural resources, emphasizing the need to implement more sustainable and responsible practices. However, the food industry has shown low R&D intensity and limited adoption of innovations, attributed to strategic barriers such as high costs and lack of cooperation [31]. 

Despite the challenges, eco-innovation in the food industry offers business opportunities and economic benefits. The development of new products, more efficient processes, and innovative conservation methods are examples of innovations that can drive the growth and competitiveness of companies [31]. Additionally, technological eco-innovation and R&D expenditure also favor firms’ performance in this sector [31].

Market attention towards sustainable and environmentally friendly practices also drives the need for eco-innovation in the food industry. Companies must address consumer expectations and respond to the demands of a market that increasingly values sustainability [32]. Open innovation, which involves learning from other companies and implementing proprietary strategies, can foster eco-innovation and improve sustainability and business performance in this industry [33].

It is essential to highlight that eco-innovation in the food industry contributes to organizations’ economic growth and addresses global challenges. The UN has recognized the importance of these practices in overcoming the challenges humanity faces in the next decade [34]. For example, the challenge of the environmental impact associated with agriculture, including soil degradation and pollution, and water scarcity [35]. In Chile, agriculture generates the most significant source of annual CO_2_ emissions and significantly contributes to global climate change [36]. Additionally, the pollutant emissions caused by food production and consumption represent a significant environmental concern. Furthermore, the European Commission on Industry 5.0 [37,38] highlights the need to involve consumers as a critical aspect to gain consumer acceptance of those changes and of the inclusion of artificial intelligence in the food production processes.

However, eco-innovation in the food industry may produce resistance to change and high associated costs. Consumers may exhibit food neophobia, a reluctance to accept and enjoy new or unfamiliar foods [39]. Furthermore, animal farming for food has been identified as one of the major causes of environmental problems, such as eutrophication, acidification, freshwater extraction, deforestation, and climate change [4].

In summary, eco-innovation in the food industry is crucial for promoting sustainability, improving business performance, and addressing environmental challenges. Despite existing barriers, implementing eco-innovative practices offers business and financial opportunities, especially for small or medium-sized companies, where attention to market expectations is a key driver for eco-innovation. 

### 1.2. Theoretical Framework: Drivers of Eco-Innovative Product Purchasing in the Food Industry

The Theory of Planned Behavior (from now on, TPB), proposed by Icek Ajzen in 1985 [16], has been used in the literature to understand human behavior and applied in numerous articles to understand healthy eating behaviors, diets, functional foods, and green foods, for example in Pandey et al. [40], McDermott et al. [41], and Menozzi et al. [42]. Even Rozenkowska [43], in her systematic literature review based on 118 peer-reviewed sources, stated a significant research tendency toward exploring consumer green behavior and the consumer purchase intention of food products under the umbrella of TPB. This theory proposed three variables that influence the formation of purchase intention: attitude, subjective norms, and perceived behavioral control. One of the primary strengths of this theory is its capacity to surpass individual intentions by incorporating the level of perceived control over behavior, and also its capacity to discern specific beliefs and attitudes that may impact the development of intentions and subsequent behavior. The theory also incorporates the differentiation between voluntary and involuntary behavior, understanding the underlying motivations behind consciously deliberate behaviors, as opposed to those that are more impulsive. Additionally, it is crucial to identify the social context and the cultural norms that can impact behavior. This enables the interpretation of external influences that may influence intentions and behaviors [44,45]. Also, this theory has overlooked a vital aspect of the purchase decision-making process related to cultural dimensions [46]. A study conducted in China adapted the TPB model to investigate the purchase intentions of eco-innovative food, considering the cultural influence on the final decision [2]. 

In the food industry, the aims to comprehend and elucidate intricate phenomena associated with food consumption and behavioral patterns [47]. For example, in the global growth of the vegan, vegetarian, and pescetarian population is a significant factor driving the orientation toward purchasing eco-innovative products. Research has identified motivators such as animal welfare, environmental care, and personal well-being, while the main barriers include sensory enjoyment associated with animal-derived products [4]. 

Within these options, certain companies within the food industry produce items that mimic animal characteristics, yet these products are crafted through entirely eco-innovative processes. For example, the Chilean company “The Not Company” utilizes plant-based technology and products to create environmentally conscious food alternatives. This development offers options that mimic textures and flavors like the animal products that people are accustomed to. 

Regarding each variable, attitude is defined as the extent to which a consumer has a positive or negative evaluation of a product, in this case, an eco-innovative food product in the food industry. Attitudinal factors have been found to explain consumer behaviors much more than, for example, sociodemographic variables such as age and income [48]. Furthermore, subjective norms refer to consumers who believe that others influence their behavior in consuming eco-innovative food products in the food industry. Finally, perceived behavioral control refers to consumers using their power to say yes or no. 

In summary, it is crucial to incorporate options in the food culture that do not originate from animal sources or cause irreparable environmental harm. Animal farming for food has been identified as one of the leading causes of environmental problems. Adopting eco-innovative practices generates market stimuli, improved operational returns, strengthened positioning, and favorable brand reception. Understanding these products’ purchase intentions and motivators is crucial for implementing metrics that promote consumption and increase the market, creating new business opportunities aligned with eco-innovation, and highlighting the importance of constant innovation in food practices to promote sustainability and environmental care. 

## 2. Materials and Methods

Research Design. Under a qualitative methodology, especially a case study, this research analyzes the purchasing motivators of consumers of eco-innovative products using “The Not Company” or NotCo. This case was selected because it exhibits eco-innovative characteristics in the food industry. Qualitative research fulfills specific conditions in the social sciences, where the authors’ orientation is crucial for developing and understanding the investigation [49]. Additionally, authors Stake [50] and Yin [51,52,53] suggest the case study as a qualitative approach that gathers information through an in-depth analysis of a particular event using various mechanisms. This study conducted semi-structured interviews to obtain diverse insights regarding consumer behavior toward NotCo. This approach offers flexibility and aligns with the research objective as questions are asked, and consumer responses are obtained. This flexibility is relevant because initial interviews may suggest additional information the analyst can gather through observation or subsequent interviews [54].

Data collection and sample. To collect data from consumers, we focused on primary sources of information. Non-probabilistic convenience sampling was used. The recruitment process was carried out using an iterative purposive sampling strategy, with the aim of including participants informed about the study topic [55,56]. According to Miles and Huberman [57], in qualitative research, participants, their purpose, and the research site should be defined. In this case, participants were defined as consumers who had purchased and consumed eco-innovative products from NotCo at least once and had consumed their products in the emerging economy of Chile, the country in which the company was launched. Semi-structured interviews were conducted regarding their purchasing and consumption experiences, utilizing a convenience sample. The number of participants reached saturation at 20 interviews. Then the Grounded Theory Approach was followed. Charmaz [58] suggests that data collection should cease when categories or themes under analysis reach saturation, meaning that gathering new data no longer generates new insights. This sample size was deemed appropriate. In qualitative methodology, there is no single and objective answer about the number of interviews necessary [59]. Participants were added to the sample until sufficient information was obtained to describe the study’s context and reach the theoretical saturation point. This saturation point was reached in interview number twenty (*N* = 20), with no additional information being obtained, which allowed the data collection process to be completed [60]. To understand the saturation point, the research question was first defined, to focus the analysis and identify when the information obtained could provide an answer to the question. Then, a scope was diagnosed, that is, from whom the information would be obtained. Once each interview was completed, it was transcribed. Next, the data analysis was carried out by organizing the information and following Braun and Clarke [17]. This analysis allowed for a continuous review and evaluation of new ideas detected in the interviews, making a contrast with respect to the interviews completed previously. Finally, when this stage is reached without major difference with respect to the data obtained previously, the saturation point is identified [49,61,62]. 

Potential interviewees were contacted via the social media platform Instagram by 3 of the authors of this study, who were previously trained to conduct the interviews. The profile @thenotco was accessed, with 187,000 followers (as of December 2022). Some followers were then approached through direct messages on Instagram, which read: “Hello, we are doing a research study; this project seeks to learn about the purchase motivators of the Not company. My mission is to contact people who have purchased or consumed any of Notco’s products to conduct an interview of approximately 30 min, which is confidential; that is, your name will not be revealed, and the information provided will only be used for academic purposes. Would you like to participate? When do you think we can meet (at the time you can) by Zoom? From already, thank you very much”. Social media are becoming a more popular means of reaching participants in social sciences research. Subsequently, a confidentiality document was sent, ensuring the anonymity of the interviews and the information provided. Once the followers read the message and agreed to participate in the interview, they were contacted via telephone and scheduled a video conference interview using the Zoom platform, version 15.6. The average duration of the interviews was approximately 16 min. In this process, it was important to prevent interviewee fatigue, which was the responsibility of the interviewer. In this context, the main mission was to remain alert to the context and cues of the interviewee; a reflexive listening was carried out, in the sense of making explicit the understanding and evaluation of the respondent’s responses. Also, active participation was sought on the part of the interviewer, posing the questions at relevant moments and seeking to maintain an appropriate pace according to the attitude of the interviewee. The questions were organized in such a way as not to have difficult questions that were demanding of the interviewee [61,62].

Table 1 presents the demographic characteristics of the sample. 

The sample consists of consumers with an average age of 25 years (ranging from 19 to 34 years), comprising 55% males and 45% females. Most participants identify as students, and 60% of the participants follow a carnivorous dietary pattern. Regarding the consumption of NotCo products, 40% of the interviewees affirm daily consumption of eco-innovative products such as NotMilk, NotMayo, and NotBurger, to name a few. 

Instrument. The questionnaire was developed to understand the purchasing motivations of NotCo products from the consumers’ perspective based on the TPB. The questionnaire consisted of two parts. The first part included questions related to sociodemographic information, personal background, and purchase frequency. The second part focused on the participants’ buying behavior, such as their perception of the environmental impact of everyday food products, including waste, animal-related issues, CO_2_ pollution, etc.

Additionally, it explored the importance of consuming the same products as their close group (family, friends, acquaintances, colleagues) and the reasons behind it. The questionnaire also investigated the factors influencing the decision to purchase NotCo products and whether participants believed they could consume NotCo products whenever they wanted. After the questionnaire was developed, it was validated by two experts in the field. Two pilot tests were conducted to assess the consistency of the questions before proceeding with the interviews. 

**Figure 1:**
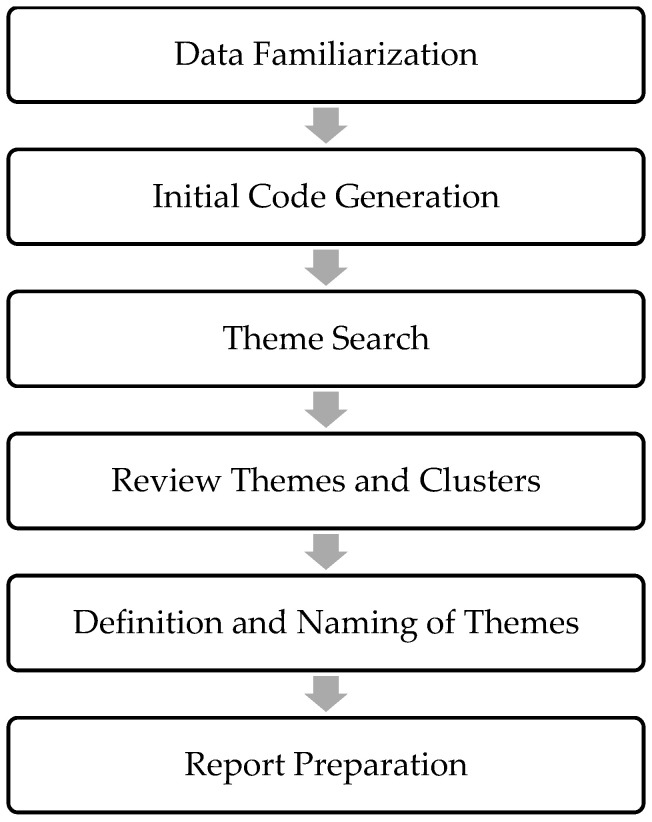
Phases for thematic analysis by Braun and Clarke [17]. Source: Elaboration from Braun and Clarke [17].

Analysis of interview data. The interviews were analyzed using the thematic analysis method proposed by Braun and Clarke [17]. This method identifies, analyzes, and reports patterns or themes within the data, organizing and describing the information. As the authors suggest, it often goes beyond description and interprets various aspects of the research topic [17]. The analysis consisted of six stages, as shown in Figure 1. The first stage involved familiarization with the data, where the interview material was organized by listening to the audio recording and transcribing them into a text editor. This process was one of the most critical activities in the research [63]. Ideas for coding were also recorded. The second stage involved generating initial codes by coding the most relevant aspects of the data, considering consumers’ meanings and their intention to purchase NotCo products in relation to sustainable development. The third stage focused on searching for themes involving the classification of codes. At this point, the authors organized the data and compared them to identify convergence. The fourth stage involved reviewing and refining the themes and their respective groupings. The fifth stage encompassed defining and naming the themes, capturing the essence of each theme, and generating subthemes based on the data related to NotCo consumption. Finally, the sixth stage involved selecting the final fragments of text to draft the research findings.

Case selection. “The Not Company” or “NotCo.” Founded in 2015 by Matias Muchnik, Pablo Zamora, and Karim Pichara, NotCo is an industrial food products company that produces plant-based alternatives to meat and dairy using AI. NotCo’s products achieve a taste, texture, and nutrition similar to animal-based products but with a more sustainable formulation, thus demonstrating a high degree of environmental and social responsibility. With the mission to “remove animals from food production without ever compromising on taste” [64], NotCo aspires to be a leading competitor in the global market for plant-based proteins, which generated USD 18.5 billion in revenue in 2019 [65].

In 2020, the World Economic Forum named NotCo one of the 100 most innovative startups worldwide. It is the only Chilean startup to receive this recognition and one of four Latin American venture companies worthy of analysis. Today, NotCo serves as an example of an innovative Chilean company aiming to become a leader in the plant-based protein market in the United States [66]. According to the company, NotCo has created not only a 100% plant-based burger but also a Whopper in partnership with major restaurant chains and the NotManjxr, a product associated with Dunkin’ Chile [67]. 

**Figure 2:**
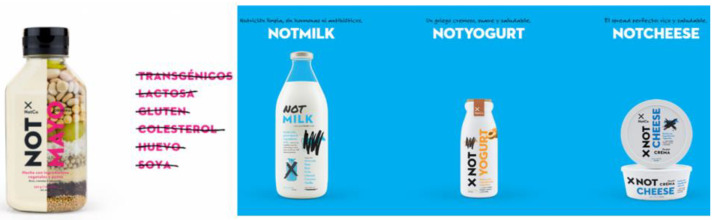
Examples of eco-innovative products from NotCo: NotMayo, NotMilk, NotYogurt, NotCheese. Source: Retrieved by CNN Chile (https://bit.ly/44a6y3O, accessed on 11 November 2022). Note: The image shows the company’s products from NotCo, and the words crossed out are ingredients excluded. Transgénicos meaning transgenic; Lactosa meaning lactose; Gluten meaning gluten; Colesterol meaning cholesterol; Huevo meaning egg; Soya meaning soy.

According to founder Muchnick, the goal is for their products to be present in at least 20% of Chilean households within five years, and today, it strengthens its path with the recent certification as part of the B company movement, focusing on governance, planet, and community [67]. This view represents a revolutionary approach to nutrition, driven by understanding what one consumes and the opportunity to create solutions to harmful manufacturing processes while considering the fusion of technology and food production as a robust alternative. Examples of the company’s products are depicted in Figure 2. 

The criteria and characteristics of this company are suitable for studying consumption behavior in Chilean society, which exhibits a higher level of environmental concern and consumer awareness compared to other countries in the region.

## 3. Results

According to the thematic analysis conducted on the 20 interviews with consumers of eco-innovative products, Table 2 presents the three perceived themes based on the TPB: sustainable attitude, subjective norms, and perceived behavioral control. Additionally, it outlines the nine subthemes: belief in food manufacturing and packaging, informed and conscious eating, against animal exploitation, adoption of lifestyle, influence of social media and advertising, willingness to ignore the preferences of others, experience with eco-innovative products, economic availability, and availability of the product in the market. These concepts can be seen in the word cloud in the Appendix A.

Firstly, regarding the “attitude” theme, referred to as “sustainable attitude”, four subthemes were identified: belief in food manufacturing and packaging, informed and conscious eating, against animal exploitation, and adoption of lifestyle. Concerning the subtheme of “belief in food manufacturing and packaging”, interviewees expressed confidence in the products they consume without any specific limitations that could harm or not harm the environment. For instance, interviewee 15, who follows a vegan diet for animal protection reasons, mentioned that she does not pay much attention to the environmental impact of the food she consumes, stating, “I’m vegan [...] I don’t consume anything from animal sources, and I don’t really pay attention to whether it harms the environment, like the packaging or things like that, I don’t focus on that” (interviewee 15). Similarly, interviewee 6 acknowledged the harmful nature of plastic packaging for the environment but stated that he still consumes products packaged in plastic because most food products are available in such packaging. She said, “Actually, for example, most packaged products are made of plastic, so the more you consume, the more you throw away and pollute [...] It’s not something that stops me from doing it” (interviewee 6).

Regarding the subtheme of “informed and conscious eating”, interviewees demonstrated a tendency towards informed and conscious consumption, focusing on understanding the processes involved in the food products they consume. Interviewee 2 stated, “*I actually feel more comfortable with ethical consumption rather than healthy consumption. I care a lot about the impact of the products I consume and try to cause the least possible harm with them [...] I’m not clear about the environmental impact of NotCo’s manufacturing processes. Still, I hope it aligns with the values we see from the company*” (*interviewee 2*). Furthermore, interviewee 3 emphasized the importance of being involved and knowing how food is sourced, stating, “*It’s very important because you need to know what’s behind what you consume [...] soybeans aren’t as healthy as one might think in terms of sustainability, and that’s when we should shift our consciousness towards what we consume, as it involves making decisions based on our own ethics*” (*interviewee 3*). Additionally, the lack of available information about the processes involved in manufacturing such foods raises concerns and acts as a barrier when deciding whether to purchase certain products, as expressed by interviewee 8: “*Ethical consumption is super important. That’s why I don’t like eating processed meats because I don’t know the process, and there’s a lot of discussion about that*” (*interviewee 8*).

Regarding the subtheme of “against animal exploitation”, interviewees expressed care for animals and a commitment to responsible consumption, aiming to minimize their impact. Interviewee 2 mentioned the possibility of reversing these situations through individual actions, stating, “*They are damaging the environment, and the food industry harms the environment. If something as simple as the way you eat can change the reality for future generations, then we should commit to that*” (*interviewee 2*). Interviewee 4 referred to the livestock industry, stating, “Because I think they abuse these animal products, it’s very cruel to kill them, and this production has become so massive that a lot of meat goes to waste [...] a lot of water is also consumed” (interviewee 4). Moreover, cultural influences exert certain pressure regarding the dietary behavior individuals are accustomed to, necessitating a degree of socio-environmental ethics when consuming food. As expressed by interviewee 13, “*I believe ethical consumption is very important, but I also think it’s a highly personal opinion [...] I hope the world could reduce its consumption of animal-derived foods and have the consciousness to abandon animal-based products, either for personal health reasons or for the environment, while empathizing with animals*” (*interviewee 13*).

Regarding the subtheme of “adoption of lifestyle”*,* in the data analysis, respondents expressed a repetitive pattern related to the available time for engaging in activities beyond their daily routines, such as sports. As indicated by interviewees 2, 7, and 18: “*It’s difficult for me to include sports in my routine; constantly changing schedules*” (*interviewee 2*). “*I wish we could all have time for sports, but sometimes or rarely can we manage it*” (*interviewee 7*). “*That’s also very important, but I don’t do it regularly because of university commitments*” (*interviewee 18*). Moreover, other interviewees, such as 6 and 20, did not express any initiatives to exercise or change their current lifestyle. Interviewee 6 stated, “*I don’t engage in it; I lack motivation, to be honest*” (*interviewee 6*). And interviewee 20 stated, “*Not at all. I’m quite lazy in that sense. I prefer watching series or going out to enjoy my free time*” (*interviewee 20*).

Secondly, regarding the “subjective norms” theme, two subthemes were identified: the influence of social media and advertising and the willingness to ignore the preferences of others. Concerning the subtheme of “influence of social media and advertising”, interviewees acknowledged the relevance of advertising in their consumption of NotCo’s eco-innovative products or similar food items. Interviewee 3 and Interviewee 6 expressed the impact of advertising on their purchasing decisions. Interviewee 3 stated, “*It affects me because when I see the advertisements, it makes me want to buy*” (*interviewee 3*). Interviewee 6 shared a similar sentiment, saying, “*I think it influences 100% because I spend a lot of time on Instagram, and I see advertisements. For example, the ‘Not Icecream’ ice cream has been around for a while, and through advertisements, I started buying it. I saw that they released a cheese, and I also felt like trying it*” (*interviewee 6*).

Regarding the subcategory of “willingness to ignore the preferences of others”, interviewees stated that their consumption choices were more individualistic and not dependent on others’ tastes. Interviewee 6, for instance, mentioned living with parents who do not consume such products but had no issue consuming them herself. She stated, “*I live with my dad and mom, and neither consumes these products. If I don’t buy them, they won’t buy them. It’s not a problem at all*” (*interviewee 6*). Similarly, interviewee 13 indicated that her household did not consume these products, but she positively encouraged their consumption, irrespective of others’ preferences. She explained, “*I mean, they consume them, but their consumption doesn’t influence me because most of the people who consume them do so because I’ve encouraged them to try it. It’s like, hey, look, try this burger, it’s delicious, it’s made from soy. But they consume them because I’ve influenced them, not because of me*” (*interviewee 13*). 

Finally, thirdly, for the theme of “perceived behavioral control”, three subthemes were identified: experience with eco-innovative products, economic availability, and availability of the product in the market. Regarding the subtheme of “experience with eco-innovative products,” respondents expressed positive aspects of the products. As interviewee 2 stated, “*Completely positive, the experiences with NotCo and its products, as I mentioned before, they are good products, and they replace the food that harms the environment. However, I’m unclear about the extent of the environmental impact due to the manufacturing of NotCo products*” (*interviewee 2*). Similarly, interviewee 6 also mentioned having a good experience with this type of food, leading to repeat purchases of the brand’s products. Interviewee 6 stated, “*I would say it’s very positive because I haven’t had any bad experiences or anything that would make me not want to buy their products so far. I mean, I would have to encounter a new brand that I like much more and has better products*” (*interviewee 6*).

Regarding the subtheme of “economic availability,” respondents mentioned the price of these food products as a determining factor in their purchase decisions, as indicated by interviewees 1, 2, and 15. Interviewee 1 stated, “*Price affects it because vegetarian products used to be even more expensive and less accessible. This situation greatly influences the decision because, without money, you can’t buy them, especially these options with different and modern nutritional styles. Someone who earns the minimum wage can’t afford these products*” (*interviewee 1*). While interviewee 2 stated, “*[…] if I have a bad month at work, obviously I won’t consume NotCo or any brand that has more expensive products that are ultimately a luxury*” (*interviewee 2*). And the interviewee 15 stated, “*price influences it because if they are going to offer new nuggets or a new burger for 7000 pesos, I’m not going to buy a burger at that price*” (*interviewee 15*).

Regarding the subtheme of “availability of the product in the market”, interviewees 2 and 15 indicated that these eco-innovative products still do not have widespread availability in various retail outlets. Interviewee 2 mentioned, “*They don’t sell NotCo near my house like I have to go to the supermarket about 5 blocks away, and I’m not always in the mood to go to a supermarket to buy it*” (*Interviewee 2*). Interviewee 15 stated, “*Stock availability obviously affects it. I only eat if it’s available in the supermarket where I regularly shop*” (*interviewee 15*). On the other hand, interviewee 5 stated that he has tried these types of products when he sees them in stock and finds them intriguing. He mentioned, “*Sometimes I define it as a ‘hunch,’ as I saw it, and I want to buy it […] I want to consume this mayonnaise because I saw it!*” (*interviewee 5*).

## 4. Discussion

Based on case study methodology and semi-structured interviews, and under the umbrella of the TPB, this study revealed various consumers’ motivations toward eco-innovative products, including attitude, subjective norms, and perceived behavioral control. Furthermore, relevant subthemes are identified, such as beliefs regarding food and packaging manufacturing, the influence of social media and advertising, and experiences with eco-innovative products and goods.

Moreover, while awareness and understanding of eco-friendly products are on the rise, eco-innovation has emerged as an opportunity for new products and markets, providing the chance to create and capture value for consumers and companies [7,68], such as seeing plant-based drinks as sustainable and nutritious alternatives to cow’s milk [13].

Therefore, this study supports previous work establishing that consumers take responsibility when selecting the products they consume daily, entering a new consumption phase characterized by increased accountability, care, and concern for the environment [69]. More specifically, the results of this research are supported by the motivations of consumers of NotCo, who consider sustainable attitudes, the influence of others in consuming eco-innovative products in the food industry, and the control over their consumption choices as relevant factors.

The present study brings some additional insights; first, this study shows that consumers who exhibit a more sustainable attitude, assuming a commitment to minimize environmental harm, display a proactive attitude towards eco-innovative products, partially in line with Coderoni and Perito [70], who described that in the absence of food neophobia, consumers are more willing to buy waste-to-value food. Our main arguments can be appreciated in respondents reducing their meat consumption, which can be associated with concerns about animal welfare, as stated in previous research [71]. Second, interviewed consumers express concerns about sourcing raw materials for food production to the manufacturing and packaging processes, highlighting their emphasis on product sustainable design, which has been described as crucial for making a significant contribution to sustainable development [72]. This concern places consumers in a divergent position from the trends observed in previous years, where their awareness was mainly focused on health issues over more general sustainability problems, leading to a prevalent demand for organic food products [73,74]. Third, consumers express that eco-innovation offers positive assurances in the commercial exchange associated with eco-innovative products in the food industry. This revelation goes in line with NotCo statements that declare the adoption of plant-based innovation, which the UN and the Food and Agriculture Organization (FAO) deem urgent for global dietary inclusion, emphasizing the importance of sustainable diets comprising locally sourced, minimally processed, and ecologically based products, in line with the findings of Sabaté and Soret [75], in a sample from India investigating individualistic norms and perceived marketplace influence on buying behavior toward eco-innovation-driven products. Fourth, an additional interesting finding highlighted in the analysis pertains to physical performance, regarding today’s sedentary lifestyles. Consumers indicate that by consuming eco-innovative products, they pay more attention to their calorie intake to compensate for the lack of exercise. This situation may be a consequence of the need to be attentive to the content and preparation of the products, which includes the recognition of the ingredients, their effects on health, and their effects on the environment.

Furthermore, it is found that a critical purchasing motivator is subjective norms. Interviewees associate this with the influence exerted by others, such as information disseminated on social media or advertising generated through platforms like Instagram, TikTok, and Facebook by the company. Previous studies have shown that social media advertising increases product awareness and positively influences customers’ engagement in pro-environmental behavior [76].

Finally, perceived control is recognized, showing that various elements either attract or deter customers from using NotCo products. Consumers, for example, note the price of the products, suggesting that they must have a sufficient monthly income to make these purchases. Rezai et al. [77] clearly state that the environment is continuously damaged, directly impacting the food chain. As a result, an increasing number of individuals are now fully embracing the green movement. In fact, customers are willing to pay more for eco-friendly products which is consistent with the behavior of eco-innovative product consumers who rely on the availability and accessibility of these products.

## 5. Conclusions

Eco-innovative products have ushered in a transformative era in the food industry, leveraging technological advancements to supplant traditional animal-based ingredients with plant-based alternatives. Our study, rooted in a comprehensive analysis of 20 semi-structured interviews, illuminates the underlying motivations driving consumers’ adoption of plant-based alternative foods within an emerging country context. These motivations coalesce into three overarching themes: sustainable attitudes, subjective norms, and perceived control. Within these thematic categories, we expound upon nine intricate subthemes, encapsulating beliefs in food manufacturing and packaging, consumer awareness and knowledge, aversion to animal exploitation, embrace of an active lifestyle, the omnipotent influence of social media and advertising, collective preferences, firsthand experiences with eco-innovative products, economic accessibility, and the presence of eco-innovative offerings in the market. This exhaustive exploration affords us a profound comprehension of the multifaceted reasons behind the consumption of eco-innovative foods.

These findings constitute a significant theoretical contribution to the marketing literature, illuminating the intricacies of consumer behavior and the dynamic landscape of sustainable product innovation. Furthermore, this study enriches the existing body of research on eco-innovation within the food industry, as it applies the TPB to emerging economies where sustainability and the pursuit of SDGs stand as paramount objectives.

Practically, our results hold interesting implications for companies operating within the food industry, particularly for innovators like NotCo, who seek to diversify their product portfolios by integrating plant-based alternatives and cutting-edge technology. This becomes especially pertinent in the backdrop of mounting concerns for environmental sustainability, necessitating judicious management of resource-intensive crop production. Therefore, our findings present insights into consumer motivations and facilitate the calibration of corporate strategies.

Nevertheless, our study recognizes certain limitations that warrant consideration. Some intricacies in food education, including ethical factors pertaining to health standards, animal welfare, and the substitution of animal-based foods, can present multifaceted aspects that may potentially influence our findings. Furthermore, the use of qualitative methodology may introduce elements of subjectivity and the potential for generalizations, as there is a possibility of bias affecting the interpretation of coding and sub-theme development. Lastly, it is important to note that our study sample is limited to residents from a single country within a specific age range, which calls for prudence when extrapolating our conclusions.

However, these limitations are not roadblocks but gateways to future research avenues, particularly centered on reflective practices. Drawing from our current findings, the deployment of quantitative questionnaires holds promise in probing the significance of motivations, their influence on behavioral intentions, and how they might be influenced by variables such as age (across diverse ranges), gender, cultural contexts, and other intervening factors. This, we believe, will drive the field forward and offer a more comprehensive understanding of the intricate web of consumer motivations within the context of eco-innovative foods.

## Figures and Tables

**Table 1:** Participant information.

I	G	Age	Occupation	Diet	Year: 1st Buy	Consumption of NotCo Product	Frequency of Purchase/Month
I1	M	26	Psychologist	Carnivorous	2019	NotBurger, NotChicken	twice/month
I2	M	24	Student	Carnivorous	2020	NotMayo, NotMilk, NotIceCream, NotMeat	daily
I3	M	22	Student	Carnivorous	2014	NotMayo, NotBurger, NotIceCream	once/month
I4	F	23	Student	Carnivorous	-	NotMayo, NotMilk, NotChicken	once/month
I5	M	30	Independent	Carnivorous	-	NotMayo	once/month
I6	F	29	Student	Carnivorous	2020	NotBurger, NotIcecream	eight times/month
I7	M	33	Student	Vegetarian	2017	NotChicken	three times/month
I8	M	28	Engineer	Pescetarian	2019	NotMayo, NotMilk, NotChicken, NotBurger	daily
I9	M	23	Independent	Carnivorous	2018	NotBurger, NotMilk	once/month
I10	F	19	Student	Vegetarian	2018	NotChicken, NotMilk, NotBurger, NotMayo	daily
I11	M	22	Student	Carnivorous	2018	Notburger, NotMayo, NotMilk	once/month
I12	F	26	Student	Carnivorous	2016	NotMayo, NotMilk	daily
I13	F	24	Student	Pescetarian	2019	Notburger, NotMayo, NotMilk	daily
I14	F	21	Student	Carnivorous	2015	NotChicken, NotMilk	daily
I15	F	23	Student	Vegan	2011	Notburger, NotMayo, NotMilk	daily
I16	F	25	Engineer	Pescetarian	2018	NotChicken, NotBurger, NotMayo, NotMilk, NotIceCream	four times/month
I17	M	22	Student	Vegan	2002	NotMayo, NotIceCream	three times/month
I18	F	24	Student	Carnivorous	2016	NotMayo, NotMilk, NotIceCream	daily
I19	M	34	Engineer	Vegetarian	2018	NotMayo, NotMilk, NotIceCream, NotMeat	four times/month
I20	M	29	Engineer	Carnivorous	2015	Notburger	daily

Source: Elaboration of the authors from the interviews carried out. Note: I: Interviewed; G: Gender.

**Table 2:** Motivations for consuming eco-innovative foods.

Themes	Subthemes
Sustainable Attitude	Belief in food manufacturing and packaging
	Informed and conscious eating
	Against animal exploitation
	Adoption of lifestyle
Subjective norms	Influence of social media and advertising
	Willingness to ignore the preferences of others
Perceived behavioral control	Experience with eco-innovative products
	Economic availability
	Availability of the product in the market

Source: Authors’ own elaboration from the interviews carried out with consumers.

## Data Availability

The data presented in this study are available on request from the corresponding author. The data are not publicly available due to the interviewers’ confidentiality, guaranteeing the participants’ privacy.

## Appendix A

Word cloud/Frequency table in Spanish and English

Coding/frequency											
Spanish	English		Spanish	English		Spanish	English		Spanish	English	
producto	product	380	gente	people	31	cotidiano	everyday	20	sentir	feel	17
alimento	food	161	hamburguesa	hamburger	30	temer	fear	20	actitud	attitude	16
consumir	consume	141	vez	time	28	colega	colleague	20	marcar	bookmark	33
notco	notco	81	alimento	food	27	tipo	type	20	not co	not co	32
sabor	taste	81	alergia	allergy	27	estar	be	19	grupo	group	25
cosa	thing	76	factor	factor	27	color	color	19	usar	use	25
impactar	impact	66	persona	person	27	vida	life	34	ganar	win	25
experiencia	experience	66	ejemplo	example	27	ruina	ruin	25	final	final	21
característica	feature	58	preciar	price	37	leche	milk	21	mayonesa	mayonnaise	21
manera	way	56	casar	marriage	26	hora	time	21	busca	search	20
carne	meat	56	dinero	money	25	desecho	waste	19	recomendar	recommend	19
animal	animal	56	hacer	make	25	medio	medium	18	respuesta	response	18
comprar	buy	56	decisión	decision	24	publicación	publication	18	consumen	consume	18
tiempo	time	55	ambiente	environment	23	realiza	performs	17	conocido	known	17
amigo	friend	50	cuál	which	22	consume	consumes	17	experto	expert	17
basar	basar	49	asociación	association	22	normal	normal	17	comportamiento	behavior	17
familia	family	48	situación	situation	22	ejercicio	exercise	33	dificultar	hinder	16
origen	origin	40	textura	texture	35	contaminación	pollution	25	control	control	16
atributo	attribute	39	red	network	26	oportunidad	opportunity	21	particular	particular	16
respecto	respect	39	dieta	diet	35	hablando	speaking	19	formar	form	16
verdad	truth	39	salud	health	26	dentro	inside	17	día	day	16
comer	eat	37	variedad	variety	24	consumidor	consumer	17			
